# Neo-adjuvant hormone therapy for non-metastatic prostate cancer: a systematic review and meta-analysis of 5,194 patients

**DOI:** 10.1186/s12957-015-0503-z

**Published:** 2015-02-22

**Authors:** Jimeng Hu, Hua Xu, Wenhui Zhu, Fei Wu, Jianqing Wang, Qiang Ding, Haowen Jiang

**Affiliations:** Department of Urology, Huashan Hospital, Fudan University, No.12 WuLuMuQi Middle Road, 200040 Shanghai, People’s Republic of China

**Keywords:** Prostate cancer, Neo-adjuvant hormone therapy, Radiotherapy, Prostatectomy, Meta-analysis

## Abstract

**Background:**

Neo-adjuvant hormone therapy (NHT) following radical prostatectomy (RP) or radiotherapy has been utilized in the multimodal approach to patients with intermediate- to high-risk prostate cancer (PCa). Herein, we performed a systematic review and meta-analysis of published randomized trials to evaluate the clinical efficacy of NHT.

**Methods:**

Literatures were searched from PubMed, EMBASE, Web of Science, and Cochrane Library for comparing neo-adjuvant therapy group (NHT plus radiotherapy or radical prostatectomy) with traditional therapy (radiotherapy or prostatectomy) alone. Quality of the research was assessed on the basis of the Cochrane’s risk of bias of randomized controlled trial. Comparable information were obtained from eligible trials and assembled for meta-analysis up to 31 August 2014. RevMan 5.2 software was used for statistical analysis.

**Results:**

Fifteen randomized controlled trials (RCTs) (total 5,194 patients) were included in this study. Meta-analysis showed there was a significant improvement in overall survival (OS) (Odds ratio (OR) = 1.51, 95% confidence interval (CI) 1.22 to 1.87, *P* = 0.0002), positive surgical margin (PSM) rate (OR = 0.30, 95% CI 0.24 to 0.38, *P* < 0.00001), and biochemical disease-free survival (bDFS) (OR = 1.95, 95% CI 1.13 to 3.39, *P* = 0.02), but no significant difference in disease-free survival (OR = 1.52, 95% CI 0.90 to 2.59, *P* = 0.12) and clinical disease-free survival (cDFS) (OR = 0.96, 95% CI 0.22 to 4.18, *P* = 0.95). Heterogeneity and risk of bias were observed between different studies.

**Conclusions:**

Patients with aggressive prostate cancer would better benefit from the receipt of neo-adjuvant therapy. Physicians should make individualized treatment strategies according to adverse reactions, financial capacities, and personal wishes.

## Background

Currently, prostate cancer (PCa) is considered to be one of the most common cancers in Western countries [1]. With the widespread use of prostate-specific antigen (PSA) screening and the development of imaging technology, PCa incidence rates have been increasing rapidly in Asian countries, especially, in developed metropolitan areas [2]. Radical radiotherapy (RT) or radical prostatectomy (RP) integrated with neo-adjuvant hormone therapy (NHT) has been utilized in multimodal treatment in patients with intermediate- to high-risk PCa.

One of the reasons why NHT is recommended for patients in all risk groups is that it can shrink prostate volume before RT or RP, thereby reducing the radiation dose to critical organs or tissue injury during operation and leading to a safer and more thorough treatment [3]. NHT plus RT or RP was found to have better efficacy than traditional therapy (radiotherapy or surgery alone) in some randomized controlled trials (RCTs), especially, in patients with intermediate- or high-risk PCa [4-6].

In trials of NHT prior to RT, specific indicators such as overall survival (OS), distant metastasis, biochemical disease-free survival (bDFS), and biochemical failure were all significantly improved compared to RT alone [7,8]. In patients receiving NHT before RP, pathological down-staging and positive surgical margin (PSM) rates were obviously improved in certain trials [9], but this did not lead to an improvement in OS and disease-free survival (DFS) [10,11].

The purpose of the present study was to determine treatment efficacy in patients who had received NHT prior to RT or RP (which is considered the first-line treatment for PCa), by performing a systematic review and meta-analysis of RCTs in men with non-metastatic PCa. In this way, we aimed to conclusively establish whether either of the treatment strategies benefited these PCa patients.

## Methods

### Literature search

This study does not involve human subjects and does not require Institutional Review Board review or consent. Articles were gathered by searching the following databases: PubMed, EMBASE, Web of Science, and Cochrane Library. In addition, abstracts and presentations were collected through major academic conferences, such as American Society of Clinical Oncology (ASCO), European Society for Medical Oncology (ESMO), and Federation of European Cancer (FEC). Furthermore, we searched the reference lists of reviews and RCTs to find potentially eligible literature. The deadline for the literature search was 31 August 2014.

We used the following MeSH terms integrated with free terms in all the search strategies: prostate cancer, neo-adjuvant hormone therapy, radiotherapy, prostatectomy, review, meta-analysis, systematic review, randomized, phase III. Only articles published in English were selected. When the study outcomes were inexplicit or more details were required, we contacted the corresponding author to obtain the original data.

### Trial identification criteria

The inclusion criteria, based on the PICO principles, are as outlined below.

(1) Participants (P): All patients who were diagnosed with non-metastatic PCa on cytological and pathological examination were eligible to be recruited to this systematic review. The nationality and race were not restricted. None of the patients had any severe concomitant disease.

(2) Interventions (I) and comparisons (C): RCTs that analyzed neo-adjuvant therapy (NHT prior to RT or RP) versus traditional therapy (RT or RP alone) in non-metastatic PCa patients, in order to compare the clinical results, including efficacy and safety, between two the groups were eligible.

(3) Outcomes (O): We included studies that compared the indicators OS, PSM, and DFS rates between the NHT plus RP group and the RP only group; and OS, bDFS, and clinical disease-free survival (cDFS) rates between the NHT plus RT group versus the RT only group.

We excluded articles if (1) the study design was not a RCT (for example, retrospective study, cohort study, and case reports), (2) the study was not an original article (for example, letters, reviews), and (3) the therapeutic effect was insufficient to obtain useful information.

### Data extraction

The information extracted from each study included the following: first author’s name, time of publication, patient characteristics, sample size, and outcomes. Data extraction was independently performed by three investigators (Hu, Xu, and Zhu). Two of the investigators (Hu and Xu) evaluated the quality of the original research by means of the Cochrane Collaboration quality checklist for RCTs, and disagreements between them were resolved by consensus.

### Data analysis

Survival rate was used as a binary variable in all the included studies. Therefore, the log of the odds ratio (OR) and the 95% confidence interval (CI) were reckoned as the effect size for each considered endpoint.

The chi-square test and *I*^2^ test were applied to detect statistical heterogeneity across trials. If heterogeneity was not present (*P* > 0.10, *I*^2^ < 50%), the fixed-effects model would be selected for further analysis; otherwise, the random-effects model would be adopted.

The OR and 95% CI results could be divided into the following: (a) OR > 1 and 95% CI not containing 1, survival rate was significantly higher in the combined therapy group than in the traditional therapy group; (b) OR < 1 and 95% CI not containing 1, survival rate was significantly higher in the traditional therapy group; (c) OR = 1, no difference in the failure rates of the two groups; and (d) 95% CI containing 1, statistically insignificant difference in the failure rates of the two groups. All statistical analyses were performed using RevMan 5.2 software.

## Results

### Study selection outcomes and quality assessment

**Figure 1:**
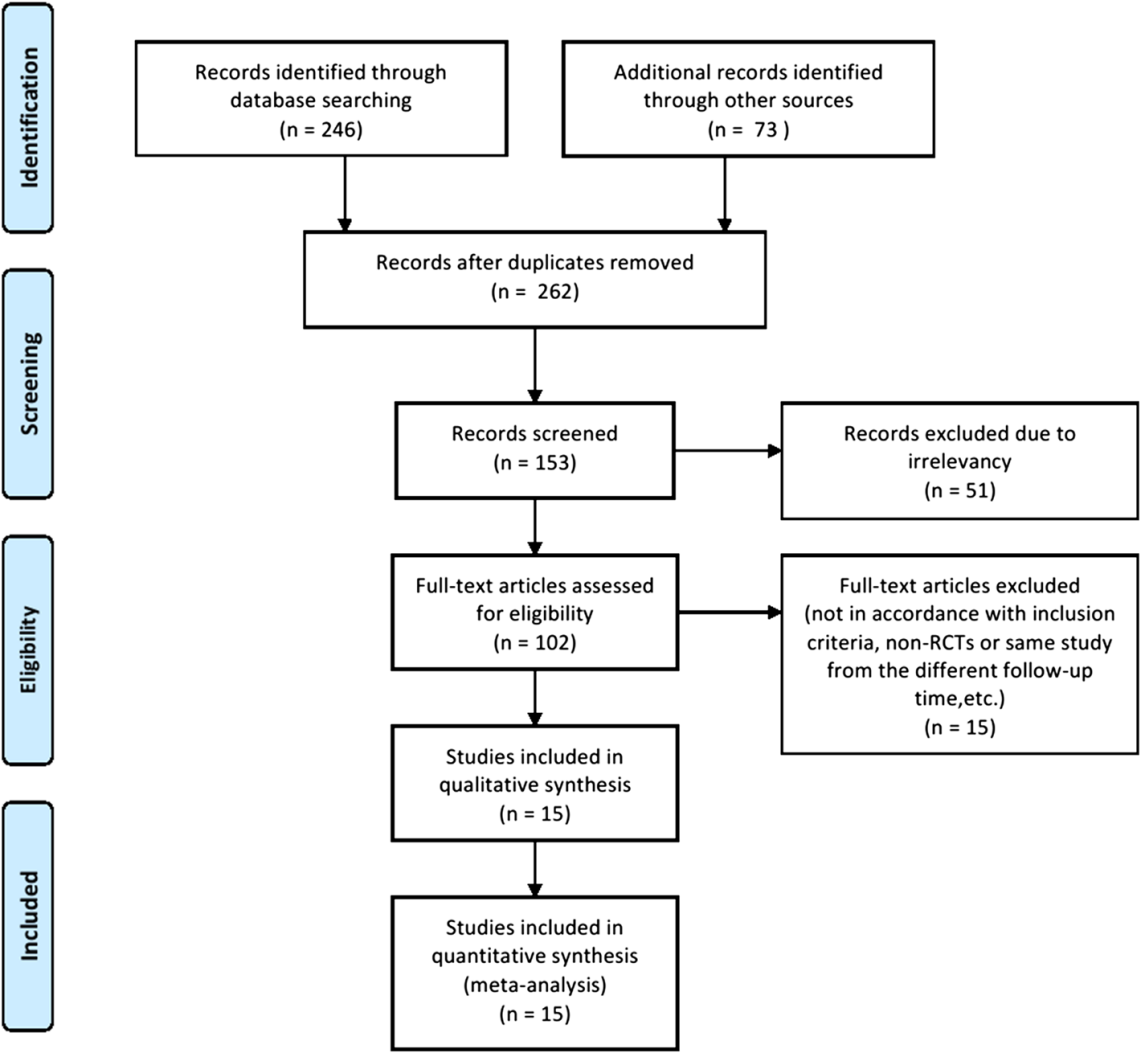
**Flow diagram illustrated the process of the study selection for the meta-analysis.**

The search strategy identified 102 possible studies from all the databases. After we eliminated studies according to the exclusion criteria, 15 papers were considered relevant to this review. The detailed literature screening process has been presented in a flowchart (Figure 1).

A total of 5,194 PCa patients were included in this study, 2,907 and 2,287 in the neo-adjuvant therapy group and traditional therapy group, respectively. The follow-up duration was not exactly the same in the included studies, but in most studies, follow-up was performed for at least 3.7 years. The pretreatment (baseline) characteristics of the patients were similar in the two groups (Table 1). The characteristics of each trial have been summarized in Table 2.

**Table 1:** **Pretreatment characteristics of patients included in systematic review**

**Author**	**Sample size (ntg/ttg)**	**Age (years) (ntg/ttg)**	**PSA level (ng/ml) (ntg/ttg)**	**Clinical stage (ntg/ttg)**	**Gleason score (ntg/ttg)**
Pilepich 2001 [12]	226/230	No record	No record	T2 to 4	2 to 6 (total: 129), 7 (total: 176), 8 to 10 (total: 124)
Laverdiere 2004 [13]	149/154	Median: 69/68	Median: 9.3/12	T2 (total: 255), T3 (total: 40)	≤6 (total: 223), 7 to 10 (total: 77)
Soloway 1995 [14]	149/154	Mean: 64.9 ± 5.7/65.4 ± 5.9	Median: 14.3/12.5	T2b, NxM0	Mean: 6.1 ± 0.17/5.8 ± 0.16
Denham 2005 [15]	270, 272/276	Median: 68, 68/67	Median: 14.4, 14.6/16.4	T2b (66, 68/72), T2c (88, 94/92), T3 to 4 (111, 105/106)	2 to 6 (117, 122/113), 7 (92, 99/114), 8 to 10 (53, 43/41)
Roach 2008 [5]	224/232	Median: 70/71	Median: 22.6/33.8	T2 to 4	3 to 6 (70/59), 7 to 10 (145/156)
Deham 2011 [6]	265, 267/270	Median: 68, 68/67	Median: 14.4, 14.5/16.4	T2b (67, 68/72), T2c (87, 94/92), T3 to 4 (111, 105/106)	2 to 6 (118, 123/114), 7 (94, 101/115), 8 to 10 (53, 43/41)
Dalkin 1996 [16]	30/31	Median: 65.5/64.7	4.1 to 10 (16/18), 10.1 to 20 (9/9), >20 (3/1)	T1c (17/16), T2a (8/12), T2b (3/0)	2 to 4 (8/6), 5 to 7 (16/21), 8 to 10 (4/1)
Goldenberg 1996 [17]	112/101	Mean: 62.5 ± 6.0/62.2 ± 5.9	0 to 4 (10/13), 4.1 to 10 (45/41), 10.1 to 25 (33/30), 25.1 to 50 (13/7)	T1b (5/4), T1c (5/3), T2a (30/33), T2b (19/17), T2c (42/34)	2 to 4 (2/5), 5 to 7 (82/75), 8 to 10 (15/11)
Labrie 1997 [18]	71/90	Range: 46 to 72	≤10 (67/53), >10 (23/18)	B0 (3/3), B1 (43/39), B2 (29/17), C1 (8/7), C2 (7/5)	No record
Schulman 2000 [9]	192/210	No record	No record	T2 (105/115), T3 (87/95)	No record
Selli 2002 [19]	143, 122/128	Mean: 65.43, 66.16/65.72	Median: 10.15, 10.0/10.20	T2 to 3, N0, M0	2 to 6 (29,2/46), 7 (31,8/1), 8 to 10 (0,11/0)
Soloway 2002 [20]	149/154	Mean: 64.9/65.4	Median: 14.3/12.5	T2b	Mean: 6.1/5.8
Aus 2002 [21]	63/63	Mean: 67/66	Median: 12.0/11.2	T1b to T1c (10/15), T2a (10/10), T2b to T3a (43/38)	2 to 4 (2/1), 5 to 6(26/22), 7 to 10 (35/40)
Klotz 2003 [10]	112/101	Median: 64/63	<10 (61/54), 10 to 20 (32/26), >20 (17/18)	T1b to T1c (12/7), T2a (36/35), T2b (19/21), T2c (41/32)	2 to 6 (75/73), 7(21/17), 8 to 10 (14/8)
Prezioso 2004 [22]	91/93	Mean: 64.9/64.5	<4 (ntg:6), ≥4 < 10 (ntg: 30), ≥10 (ntg: 39)	T1a to T2b	No record

Ntg/ttg, neo-adjuvant therapy group/traditional therapy group; PSA, prostate-specific antigen.

**Table 2:** **Characteristics of agent trials included in systematic review**

**Author**	**Inclusion criteria**	**Exclusion criteria**	**Dose (NHT)**	**Interventions**	**Follow-up time**
Pilepich 2001 [12]	Tumor size was measured by the surface area palpable by rectal examination. Performance score (KPS) = 60 positive lymph nodes if below the common iliac level	Patients with involved common peri-aortic or iliac lymph node	2 months goserelin acetate ((3.6 mg every 4 weeks) flutamide (250 mg tid)	NHT followed by RT and continued during RT versus RT alone. Pelvis: 44 to 46 Gy, Prostate: 65 to 70 Gy, 33 fractions/6.5 weeks	Median: 6.7 years
Laverdiere 2004 [13]	Age <75 years, PSA <50 mg/ml, without bone metastases	No previous hormonal therapy or chemotherapy	3 months leuprolide (7.5 mg monthly) flutamide (250 mg tid)	NHT followed by RT versus RT alone. Pelvis: 64 Gy, 32 fractions/6.5 weeks	Median: 3.7 years
Soloway 1995 [14]	Age <75 years, PSA <50 ng/ml, normal bone scan	No previous hormonal therapy or chemotherapy	2 weeks leuprolide (7.5 mg monthly) flutamide (250 mg tid)	NHT followed by RP versus RP alone	Median: unknown
Denham 2005 [15]	No bone metastases, prostatic acid phosphatase <1.8 u/ml, PSA <50 ng/ml	Renal dysfunction, hepatic disease, other malignancies or concomitant anti-androgenic medication	3 months cyproterone acetate (300 mg daily for 12 weeks), 6 months cyproterone acetate	3 or 6 months NHT followed by RT versus RT alone. Prostate/seminal vesicles: 66 Gy, 33 fractions/6.5 to 7 weeks	Median: 5.9 years
Roach 2008 [5]	bulky (5*5 cm) tumors, with or without pelvic lymph node involvement	no follow-up data	2 months flutamide (250 mg tid), goserelin (3.6 mg every 4 weeks)	NHT followed by RT versus RT alone. Regional lymphatics: 44 to 46 Gy prostate: 65 to 70 Gy	Median: 11.9 years
Deham 2011 [6]	Histologically confirmed, informed consent	Significant intercurrent medical conditions, prior malignancies or metastases	Goserelin (3.6 mg given subcutaneously every month), flutamide (250 mg tid)	3 or 6 months NHT followed by RT versus RT alone. Prostate and seminal vesicles: 66 Gy, 33 fractions/6.5 to 7 weeks	Median: 10.6 years
Dalkin 1996 [16]	PSA >4.0 ng/ml, projected survival >10 years	No record	3 months goserelin (s.c 3.6 mg monthly)	NHT followed by RP versus RP alone.	Median: unknown
Goldenberg 1996 [17]	Histologically confirmed, prostatic acid phosphatase <1.8 u/ml, PSA <50 ng/ml	Renal dysfunction, hepatic disease, other malignancies or concomitant anti-androgenic medication	Cyproterone acetate (300 mg daily for 12 weeks)	NHT followed by RP versus RP alone	Median: unknown
Labrie 1997 [18]	Histologically confirmed, life expectancy >10 years	No record	3 months flutamide and leuprolide acetate	NHT followed by RP versus RP alone	Median: unknown
Schulman 2000 [9]	Histologically confirmed, PSA <100 ng/ml	No record	3 months goserelin (3.6 mg subcutaneously depot injection each month) flutamide (250 mg tid)	NHT followed by RP versus RP alone	Median: 4.0 years
Selli 2002 [19]	Histologically confirmed, informed consent	No record	Goserelin (3.5 mg subcutaneously depot injection each month) bicalutamide (50 mg/day)	3 or 6 months NHT followed by RP versus RP alone	Median: unknown
Soloway 2002 [20]	Age <75 years, PSA <50 ng/ml, normal bone scan	No previous hormonal therapy or chemotherapy	3 months leuprolide (7.5 mg monthly) flutamide (250 mg tid)	NHT followed by RP versus RP alone	Median: 5.0 years
Aus 2002 [21]	Previously untreated, age <75 years, life expectancy >10 years	Positive lymph nodes	3 months triptorelin (3.75 mg i.m. monthly)	NHT followed by RP versus RP alone	Median: 82 months
Klotz 2003 [10]	Histologically confirmed, prostatic acid phosphatase <1.8 u/ml, PSA <50 ng/ml	Renal dysfunction, hepatic disease, other malignancies or concomitant anti-androgenic medication	3 months cyproterone acetate (300 mg daily for 12 weeks)	NHT followed by RP versus RP alone	Median: 6.0 years
Prezioso 2004 [22]	Lifespan >5 years, WHO performance status up to 2, no evidence of metastases, informed consent	No previous hormonal therapy or chemotherapy, no previous orchidectomy or other malignancies	3 months leuprolide (3.75 mg) cyproterone acetate (300 mg daily for 3 weeks)	NHT followed by RP versus RP alone	Median: unknown

NHT, neo-adjuvant hormone therapy; PSA, prostate-specific antigen; RP, radical prostatectomy; RT, radiotherapy; Gy, gray.

Although all the included studies were RCTs, only three mentioned the procedure of randomization. Two were randomized mainly on the basis of phone calls [10,16], and one was randomized by the minimization technique after stratification [6]. Only one study mentioned the number and causes of dropouts and withdrawals [13]. None of the trials described the procedures used to evaluate the results or blind the allocation of interventions.

### Overall survival

**Figure 2:**
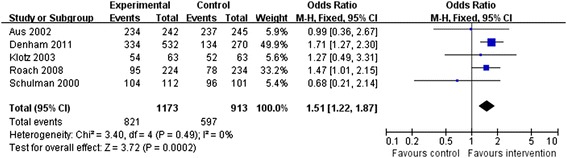
**Meta-analysis of overall survival compared neo-adjuvant therapy group versus traditional therapy group.**

Five studies evaluated OS in the neo-adjuvant therapy group compared with the traditional therapy group. Since no heterogeneity was found among these studies (*P* = 0.49, *I*^2^ = 0%), a fixed-effects model was selected for data analysis. The outcomes suggested that OS was significantly longer in the neo-adjuvant therapy group than in the traditional therapy group (OR = 1.51, 95% CI: 1.22 to 1.87, *P* = 0.0002; Figure 2).

Disease-specific survival was presented in two of the above studies [9,21] and showed no obvious improvement with neo-adjuvant therapy (relative risk = 1.00, 95% CI: 0.98 to 1.03, *P* = 0.77).

### Positive surgical margin rate

**Figure 3:**
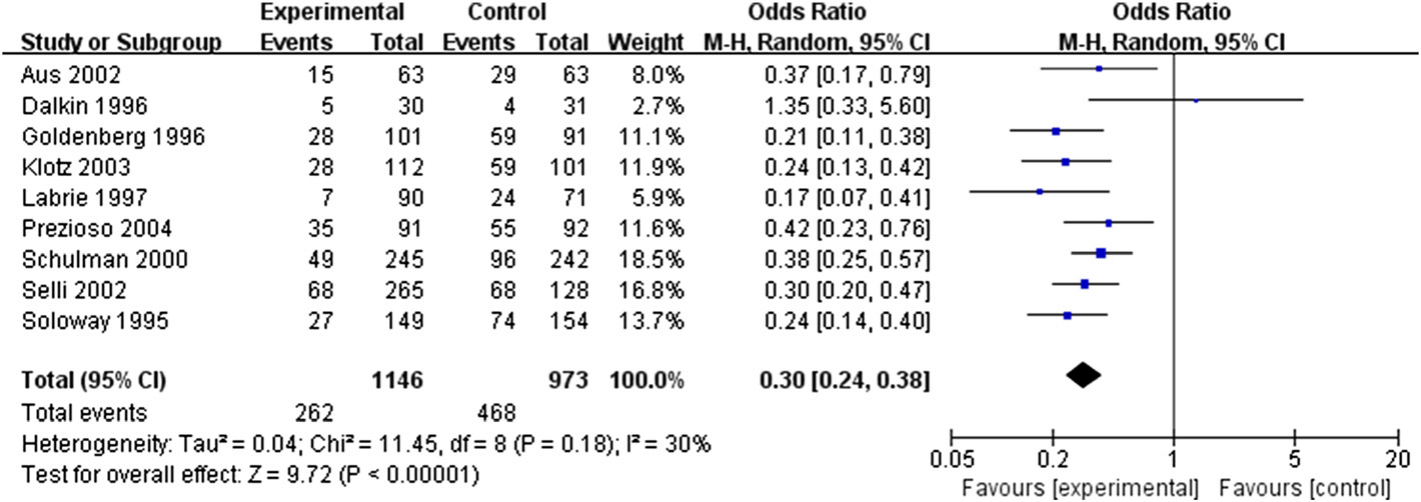
**Meta-analysis of positive surgical margin rate compared neo-adjuvant therapy group versus traditional therapy group.**

Nine RCTs assessed the PSM rates. No obvious heterogeneity was observed between these studies (*P* = 0.18, *I*^2^ = 30%), so a fixed-effects model was applied to analyze the effect size. The PSM rate was significantly lower in the neo-adjuvant therapy group than in the traditional therapy group (OR = 0.30, 95% CI: 0.24 to 0.38, *P* < 0.00001; Figure 3).

Positive lymph node involvement was found in five trials [9,10,16,20,22], and the outcome was less frequent in the neo-adjuvant therapy group (relative risk = 0.66, 95% CI: 0.47 to 0.94, *P* = 0.02).

### Disease-free survival

**Figure 4:**
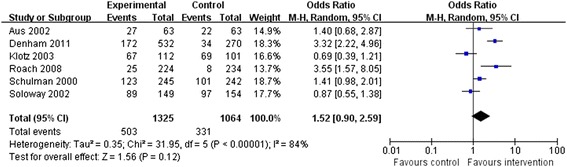
**Meta-analysis of disease-free survival compared neo-adjuvant therapy group versus traditional therapy group.**

Six RCTs reported DFS rates, and significant heterogeneity was detected among them (*P* < 0.00001, *I*^2^ = 84%). Hence, a random-effects model was chosen. There was no significant difference between the neo-adjuvant therapy group and the traditional therapy group in terms of DFS (OR = 1.52, 95% CI: 0.90 to 2.59, *P* = 0.12; Figure 4).

One study [22] attempted to assess the efficacy of NHT on PSA relapse and pathological variables in contrast with prostatectomy alone, and defined PSA relapse based on the available data. However, the median follow-up time (<7 months) was too short to meet the study requirements, and thus, the information was regarded as insufficient to accomplish the statistical analysis.

### Biochemical disease-free survival

**Figure 5:**
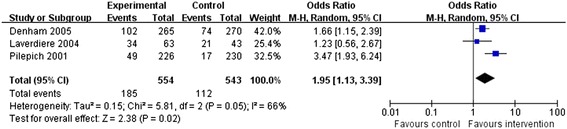
**Meta-analysis of biochemical disease-free survival compared neo-adjuvant therapy group versus traditional therapy group.**

Three RCTs examined bDFS rates. Due to apparent heterogeneity between two of these studies (*P* = 0.05, *I*^2^ = 66%), a random-effects model was adopted. In the neo-adjuvant therapy group, bDFS was significantly increased compared with that in the traditional therapy group (OR = 1.95, 95% CI: 1.13 to 3.39, *P* = 0.02; Figure 5).

Specifically, Pilepich *et al*. [12] reported the 8-year bDFS rates in the treatment (24%) and control arms (10%, *P* < 0.0001). Laverdiere *et al*. [13] found that the 7-year bDFS was significantly higher in the neo-adjuvant treatment arm than in the control arm (*P* = 0.009). Denham *et al*. [15] reported a significant improvement in the 5-year bDFS in the neo-adjuvant therapy group (*P* = 0.002).

### Clinical disease-free survival

**Figure 6:**
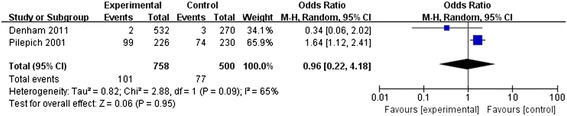
**Meta-analysis of clinical disease-free survival compared neo-adjuvant therapy group versus traditional therapy group.**

Two RCTs of 1,258 PCa patients evaluated cDFS rates in this meta-analysis. Significant heterogeneity was detected between the two studies (*P* = 0.09, *I*^2^ = 65%), and so, a random-effects model was applied. No significant difference was observed with respect to cDFS between the two groups (OR = 0.96, 95% CI: 0.22 to 4.18, *P* = 0.95; Figure 6).

## Discussion

Two meta-analyses have assessed the use of neo-adjuvant therapy for localized PCa [23,24], but the trial identification criteria and primary endpoints in these articles are different from ours. Our research demonstrated a sufficient assessment of the clinical outcomes in the neo-adjuvant therapy group compared with the traditional therapy group, in patients with non-metastatic PCa.

Although the number of relevant studies was not enough to provide a reliable scientific basis, the clinical outcomes presented herein strengthen the role of NHT combined with RT or RP for non-metastatic PCa.

### Primary disclosures

The results from the statistical analyses demonstrated that OS, PSM, and bDFS rates were significantly improved in the neo-adjuvant therapy group. These positive results suggested that NHT could considerably improve pathological outcomes and biochemical recurrence indicators such as PSM rate and serum PSA level. This shows that these treatment outcomes, which include pathological and biochemical indicators, are effective alternatives for efficacy evaluation. However, there was no significant difference in DFS and cDFS rates between the two groups in this systematic review. Considering the prognostic factors for systemic progression, Stewart *et al*. [25] found that the Gleason grading system indicates the risk of systemic progression in patients treated with prostatectomy after NHT.

Comprehensive statistical analysis revealed obvious heterogeneity between most individual studies. In our opinion, the heterogeneity may be attributable to the following factors: different types and doses of drugs, deviation in hormone allocation, definitions of biochemical recurrence, and variations in patient characteristics and follow-up duration. For instance, Laverdiere *et al*. [13] defined biochemical recurrence as two consecutive increases in serum PSA level of at least 1.0 and ≥1.5 ng/ml, according to the Vancouver rules. In contrast, Denham *et al*. [15] used the Houston method, which defines biochemical failure as increases in PSA level of 2 ng/ml or more above the post-treatment nadir measure.

This meta-analysis demonstrated that neo-adjuvant therapy plus RP or RT was associated with longer OS compared with RP or RT alone, in the overall combination therapy group as well as in each combination treatment subgroup. Furthermore, the benefits of neo-adjuvant therapy plus RP or RT included a significant enhancement of loco-regional control in patients with non-metastatic, intermediate- to high-risk PCa.

The disaggregated analysis of different risk groups indicated that the superiority of neo-adjuvant therapy was most manifest in patients with high-risk PCa. However, the clinical significance of these outcomes is restricted by the use of different risk categories among the studies. The reliability of our preliminary findings will be verified by the results of further targeted studies in this specific area.

### Risk of bias evaluation

Since about half of the researches had not offered sufficient evidence to verify whether the risk of bias in the trial identification criteria and randomization measures was low or high, the quality of evidence analyzed in this review varied.

Furthermore, it was unclear whether the outcome inspectors had been blinded to the group information in all the studies, and some authors failed to report all meaningful results, so the outcomes may be subject to reporting bias, with missing information potentially leading to an overvaluation of the observed effects.

### Limitations and prospects

The survival analyses presented in the published articles tended to support NHT in combination with RP or RT. However, many uncertainties still exist. Although OS was obviously improved in these trials, which is a tangible clinical outcome and an apparent superiority of the regimen, the value of prolonged bDFS remains uncertain and may not translate into certain survival benefit. Owing to a ‘duration of risk’ effect, it seemed that PCa patients must survive for more than 7 years to benefit from the combined treatment. All the RCTs included in this meta-analysis were from Western countries rather than Asia. Thus, whether these outcomes can be extrapolated to Asian patients must be verified by further research.

Therapeutic benefit and side effects should be balanced by evidence-based adoption of the current information. The various indicators that need to be noted include PCa grade, risk group division, sexuality, life expectancy, physical function, and endocrine/metabolic status (including hypertension, adiposity, and diabetes mellitus).

Luteinizing hormone-releasing hormone agonist (LHRHa) analogues were applied in most neo-adjuvant setting trials, and the role of peripheral anti-androgens is still unclear. Newly developed drugs acting through androgen suppression, such as enzalutamide and abiraterone, will play an important role in the future. Similarly, these drugs are considered to offer more effective androgen deprivation against prostate tumor cells, but whether they can lead to an obvious therapeutic benefit, such as tumor growth control and/or toxicity reduction, requires further comprehensive and systematic research. A recently published RCT [26] illustrated that LHRHa plus abiraterone acetate (AA) treatment could suppress tissue androgens more effectively than LHRHa alone. Thus, intensive intratumoral androgen suppression with LHRHa plus AA before RP may reduce tumor burden in patients with localized high-risk PCa.

In addition, the above agents should be assessed with regard to quality of life, adverse effects, and medical burden to the patients, these parameters could not be subjected to correlation analysis in this review. Physicians should make reasonable treatment strategies for PCa patients according to the patients’ physical conditions, financial capacities, and personal wishes.

## Conclusions

The outcomes of this meta-analysis indicated that patients with intermediate- to high-risk PCa would benefit more from NHT plus RT/RP than from RT/RP alone, although several limitations need to be addressed due to the small number of studies included.

Adverse reactions, medical costs during NHT, and other objective limitations will affect the treatment process significantly. Thus, physicians have to consider all these issues when formulating individualized treatment strategies.

## Abbreviations

AA: abiraterone acetate
ASCO: American Society of Clinical Oncology
bDFS: biochemical disease-free survival
BF: biochemical failure
C: comparisons
cDFS: clinical disease-free survival
CI: confidence intervals
DFS: disease-free survival
DM: distant metastasis
ESMO: European Society for Medical Oncology
FEC: Federation of European Cancer
I: interventions
LHRHa: luteinizing hormone-releasing hormone agonist
NHT: neo-adjuvant hormone therapy
O: outcomes
OR: odds ratio
OS: overall survival
P: participants
PCa: prostate cancer
PSA: prostate-specific antigen
PSM: positive surgical margin
RCTs: randomized controlled trials
RP: radical prostatectomy
RT: radical radiotherapy
